# Genome-wide identification and differentially expression analysis of lncRNAs in tilapia

**DOI:** 10.1186/s12864-018-5115-x

**Published:** 2018-10-04

**Authors:** Bi Jun Li, Dan Li Jiang, Zi Ning Meng, Yong Zhang, Zong Xian Zhu, Hao Ran Lin, Jun Hong Xia

**Affiliations:** State Key Laboratory of Biocontrol, Institute of Aquatic Economic Animals and Guangdong Provincial Key Laboratory for Aquatic Economic Animals, College of Life Sciences, Sun Yat-Sen University, Guangzhou, 510275 People’s Republic of China

**Keywords:** Tilapia, lncRNA, RNAseq, Network, Stress

## Abstract

**Background:**

Long noncoding RNAs (LncRNAs) play important roles in fundamental biological processes. However, knowledge about the genome-wide distribution and stress-related expression of lncRNAs in tilapia is still limited.

**Results:**

Genome-wide identification of lncRNAs in the tilapia genome was carried out in this study using bioinformatics tools. 103 RNAseq datasets that generated in our laboratory or collected from NCBI database were analyzed. In total, 72,276 high-confidence lncRNAs were identified. The averaged positive correlation coefficient (*r_mean* = 0.286) between overlapped lncRNA and mRNA pairs showed significant differences with the values for all lncRNA-mRNA pairs (*r_mean* = 0.176, *z* statistics = − 2.45, *p* value = 0.00071) and mRNA-mRNA pairs (*r_mean* = 0.186, *z* statistics = − 2.23, *p* value = 0.0129). Weighted correlation network analysis of the lncRNA and mRNA datasets from 12 tissues identified 21 modules and many interesting mRNA genes that clustered with lncRNAs. Overrepresentation test indicated that these mRNAs enriched in many biological processes, such as meiosis (*p* = 0.00164), DNA replication (*p* = 0.00246), metabolic process (*p* = 0.000838) and in molecular function, e.g., helicase activity (*p* = 0.000102) and catalytic activity (*p* = 0.0000612). Differential expression (DE) analysis identified 99 stress-related lncRNA genes and 1955 tissue-specific DE lncRNA genes. MiRNA-lncRNA interaction analysis detected 72,267 lncRNAs containing motifs with sequence complementary to 458 miRNAs.

**Conclusions:**

This study provides an invaluable resource for further studies on molecular bases of lncRNAs in tilapia genomes. Further function analysis of the lncRNAs will help to elucidate their roles in regulating stress-related adaptation in tilapia.

**Electronic supplementary material:**

The online version of this article (10.1186/s12864-018-5115-x) contains supplementary material, which is available to authorized users.

## Background

Long non-coding RNAs (lncRNAs) are transcripts longer than 200 nt but without coding potential. They play roles in transcriptional regulation and post-transcriptional regulation, and show functions in protein localization, telomere replication, and RNA interference [1–4] and conservation in Vertebrates [5]. With the development of whole genome RNA sequencing (RNA-Seq) technique and computational analysis, genome-wide identification of lncRNAs have been performed in many species, such as yeast [6], fruit fly [7], Atlantic salmon [8], zebrafish [9], chicken [10], rat [11] and human [12].

LncRNAs were closely associated with stress responses in animals. Ambient stressors, e.g., chemistry treatment [13–15], drug reaction [16], high salt [17] and pathogen infection [8] were found to induce lncRNA expression changes. For example, in mice, lncRNA TUG1 exerted a protect effect against cold-induced liver damage by inhibiting apoptosis [18].

A lot of studies have paid attention to characterize the transcriptional responses to cold stress in fishes such as common carp (*Cyprinus carpio*) [19], zebrafish (*Danio rerio*) [20–22], channel catfish (*Ictalurus punctatus*) [22], killifish (*Austrofundulus limnaeus*) [23], coral reef fish (*Pomacentrus moluccensis*) [24] and rainbow trout (*Oncorhynchus mykiss*) [25]. Gene expression changes in response to hypoxia were explored in some teleosts, such as in divergent tissues of adults of the euryoxic gobiid fish *Gillichthys mirabilis* [26], the gills [27] and the heart [28] of zebrafish and zebrafish embryos [29], the heart and liver in the teleost fish *Fundulus grandis* [30] and in Tilapia [31, 32]. However, there was very limited stress-related work on lncRNAs in economic fish. In rainbow trout [33] and Atlantic salmon [8], differential expressions of lncRNAs in response to infection were reported.

Tilapia is one of the most important cultural fish worldwide with great economic importance [34]. In tilapia, much attention has been paid to stress responses. The gene expression changes in response to different stressors including cold stress [35], alkalinity stress [36], salinity adaptation [37], hypoxia stress [31, 32, 38] have been investigated by RNA-seq. The QTL intervals for hypoxia and salt tolerance traits were mapped recently [39, 40]. Function of miRNAs was investigated under high-salt, alkalinity, hypoxia, *Streptococcus agalactiae* infection and other adverse conditions [41–48]. LncRNAs were relatively new class of RNA molecules that were less well characterized in tilapia. Recently, Hezroni et al. identified lncRNAs based on one tilapia RNAseq dataset and evaluated the conservation of lncRNAs in vertebrates [5]. The expression profile of 797 putative non-coding transcripts in tilapia macrophages activated by HSP70 and *Streptococcus agalactiae* antigen was reported [49].

In this study, to uncover lncRNA characteristics in tilapia genome, we generated and collected a total of 103 RNA-seq samples and carried out a genome-wide identification of lncRNAs. The potential association of the lncRNAs with different stressors in different tissues was analyzed. Our finding provided an invaluable resource for further studies on molecular basis of lncRNAs in tilapia.

## Results

### Genome-wide identification of lncRNAs in tilapia

Understanding the characteristics of lncRNAs in tilapia genome would be useful for exploring functional mechanisms related to economic traits. To obtain a full annotation of lncRNAs in tilapia genome, we totally generated 32 RNAseq datasets in our laboratory and collected 71 RNAseq datasets from NCBI SRA database (Additional file 1: Table S1). These samples originated from at least 15 different tissues and different stress conditions, e.g. hypoxia, salinity, Fadrozole, lipid content, alkalinity, temperature, *Streptococcus agalactiae* infection or show differences in other economic traits, such as body colors. This dataset represents the largest data collection for identification of lncRNAs in tilapia.

**Fig. 1:**
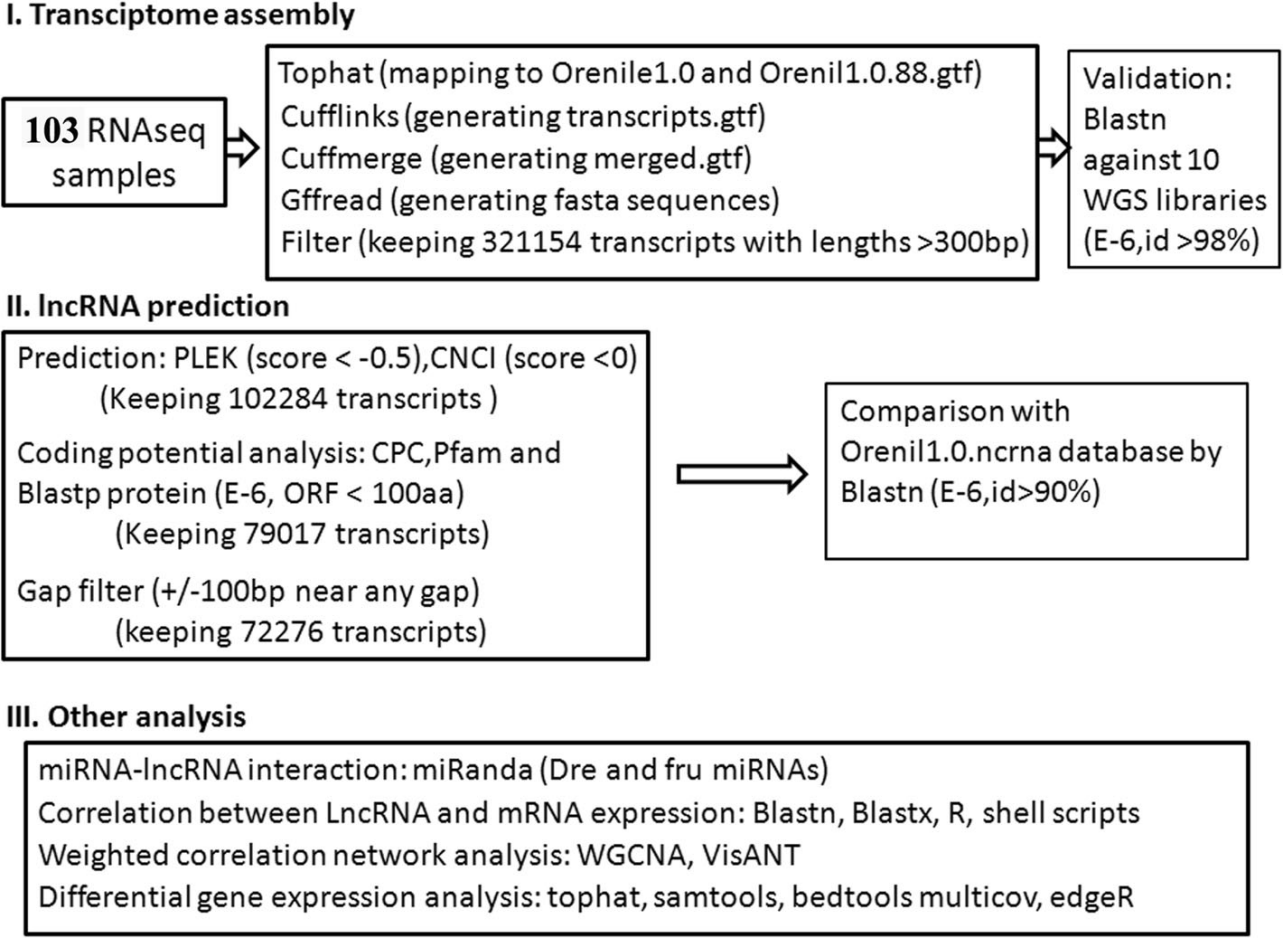
The flow chart for identification of lncRNAs in the study

A step-wise protocol was applied to ensure a high-confident discovery of lncRNAs (Fig. 1). The libraries were first mapped to the reference tilapia genome (Orenile1.0) and then the successfully mapped high quality reads were used for transcriptome assembly. The de novo assembly of the 103 RNAseq data produced 321,154 transcripts with a length more than 300 bp.

To validate the assembly, all transcripts were searched against 10 tilapia Transcriptome Shotgun Assembly (TSA) databases by using Blastn (Additional file 1: Table S1). The TSA datasets contained 1,189,903 sequences with a minimum length of 200 bp to a maximum length of 27,441 bp. The average sequence length was 1111 bp. The search indicated 186,333 transcripts in our assembly showing high similarity and an average coverage of 58% to 105,960 transcripts in the TSA datasets. The remaining transcripts with no significant hits in the TSA databases suggested lots of novel transcripts found in our dataset.

From the assembly, 102,284 lncRNA candidates were initially predicted. Further coding potential analysis suggested that 79,017 of the transcripts were candidate lncRNAs. The candidates near gaps in the genome might represent truncated coding genes. We further filtered the lncRNA candidates that located within 100 bp of the downstream and upstream of gaps. The remaining 72,276 transcripts were denoted as lncRNAs in this study (Table 1).

**Table 1:** Summary statistics for tilapia lncRNA dataset identified in this study

Item	Statistics value
Total sequences	72,276
Total bases	55,238,101
Min sequence length	300
Max sequence length	7783
Average sequence length	764
Median sequence length	669
N25 length	1167
N50 length	832
N75 length	607
N90 length	460
N95 length	395
As	28.90%
Ts	28.78%
Gs	21.11%
Cs	21.21%
Total transcripts with 1–10 SSR repeat units	4788
2 nucleotides	3638
3 nucleotides	218
4 nucleotides	606
5–10 nucleotides	926

The lengths for these transcripts ranged from 301 bp to 7783 bp with an average sequence length of 764 bp, a N50 of 832 bp and total bases of 55,238,101 (Table 1 and Additional file 2: Figure S1). The exon numbers for lncRNAs ranged from one to eight exons with an average of 1.1. Most of the lncRNAs (~ 94%) were single-exon types. The average exon number for all transcripts in the de novo assembly was 8.9 with a range from 1 to 219 exons.

Microsatellite instability is a sign of DNA mismatch repair deficiency that can be inherited, which is involved in regulation of lncRNA gene function, e.g. CCAT2 in colorectal cancer [50]. We observed 4788 lncRNAs contained 1-10 bp microsatellite repeat units. 3638 of the microsatellites were classified to the category of 2-bp repeat units (Table 1). This data will be helpful for exploring the function of microsatellite-contained lncRNAs in fish.

Identifications of the lncRNAs against the predicted 6455 noncoding RNA data (Orenil1.0.ncrna downloaded from Ensemble database) using Blastn, we found that 3263 Ensemble noncoding RNAs showed high similarity to 2532 lncRNAs in this study (Identity > 90%, <E-6) with a mean coverage length of 709 bp. This suggests a lot of noncoding RNAs have been captured in our data.

### Co-expression analysis between lncRNAs and mRNAs

LncRNAs were generally co-expressed with their neighboring genes [51]. To investigate the relationship between lncRNAs and mRNA genes in tilapia genome, we first identified mRNA candidates from the uncertain transcripts in the assembly by searching against the mRNA database (GCF_001858045.1_ASM185804v2_rna) and protein database (Oreochromis_niloticus.Orenil1.0.pep.all.fa; ID> 99%, E0). Finally 42,634 transcripts were considered to be mRNA candidates for further analysis. This may be lowly representative of the mRNAs in our dataset.

The position data of lncRNAs (4596) and mRNA candidates (42,634) with strand information that generated in the gtf file by Cuffmerge were retrieved. The gene expression data for all samples were used for correlation analysis. The average gene expression value across the 103 samples was 8322 normalized read counts for mRNAs and 1800 normalized read counts for lncRNAs. These suggest the lncRNAs were much lower expressed compared to the mRNAs, similar to previous reported such as in cashmere goat [52].

**Fig. 2:**
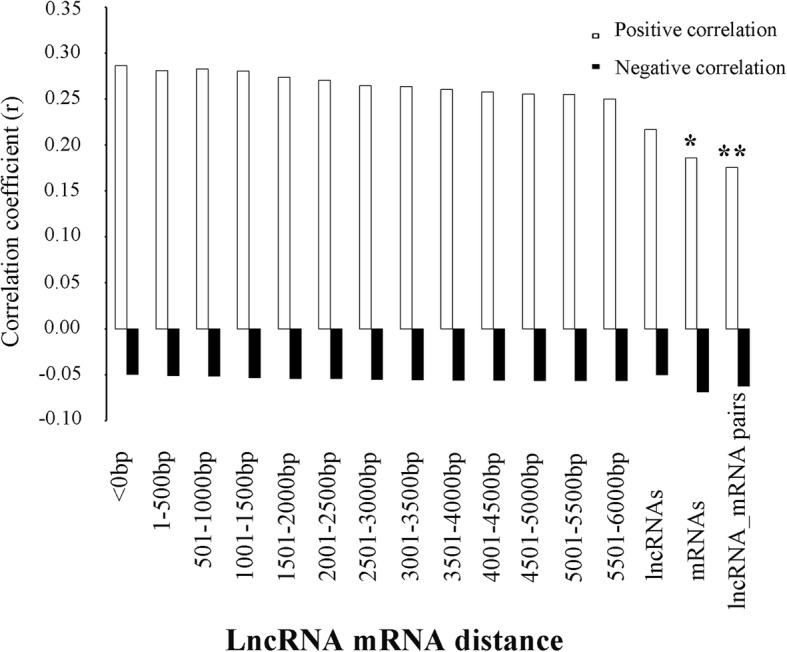
The relationship between positive and negative correlation coefficients (*r_mean*) and lncRNA-mRNA distances. The positive and negative *r_mean* for all lncRNAs, mRNAs and lncRNA-mRNA pairs were plotted. ‘*’ and ‘**’, indicate significant differences (*p* < 0.05 or *p* < 0.001) of positive *r_mean values* compared to the *r_mean* of overlapped lncRNA-mRNA pairs as assessed using the Fisher r-to-z transformation statistics

The mean negative correlation coefficient (*r_mean*) was ranging from − 0.049 to − 0.069 for all pairs and showed no significant differences (Fig. 2). We observed that the mean positive correlation coefficient (*r_mean*) was 0.286 for 444 lncRNA-mRNA pairs that overlapped with at least one basepair. This *r_mean* value was generally larger than the values for non-overlapped lncRNA-mRNA data (e.g., from 0.281 to 0.250 for lncRNA-mRNA pairs within 1-500 bp to 5500-6000 bp distances) and showed significant differences with the values for all lncRNA-mRNA pairs (*r_mean* = 0.176, *z* statistics = − 2.45, *p* value = 0.00071) and all mRNA-mRNA pairs (*r_mean* = 0.186, *z* statistics = − 2.23, *p* value = 0.0129) (Fig. 2).

To identify functional gene modules across different tissues, we randomly selected 36 RNAseq samples that originated from 12 different tissues (three samples per tissue). The lncRNA-mRNA pairs with high correlation coefficient (|r| > 0.7) including 3844 mRNAs and 1255 lncRNAs were kept for weighted Gene Co-expression network analysis (WGCNA). The cluster analysis of the lncRNA expression indicated that most samples were grouped according to the origins of tissues (Additional file 3: Figure S2).

**Fig. 3:**
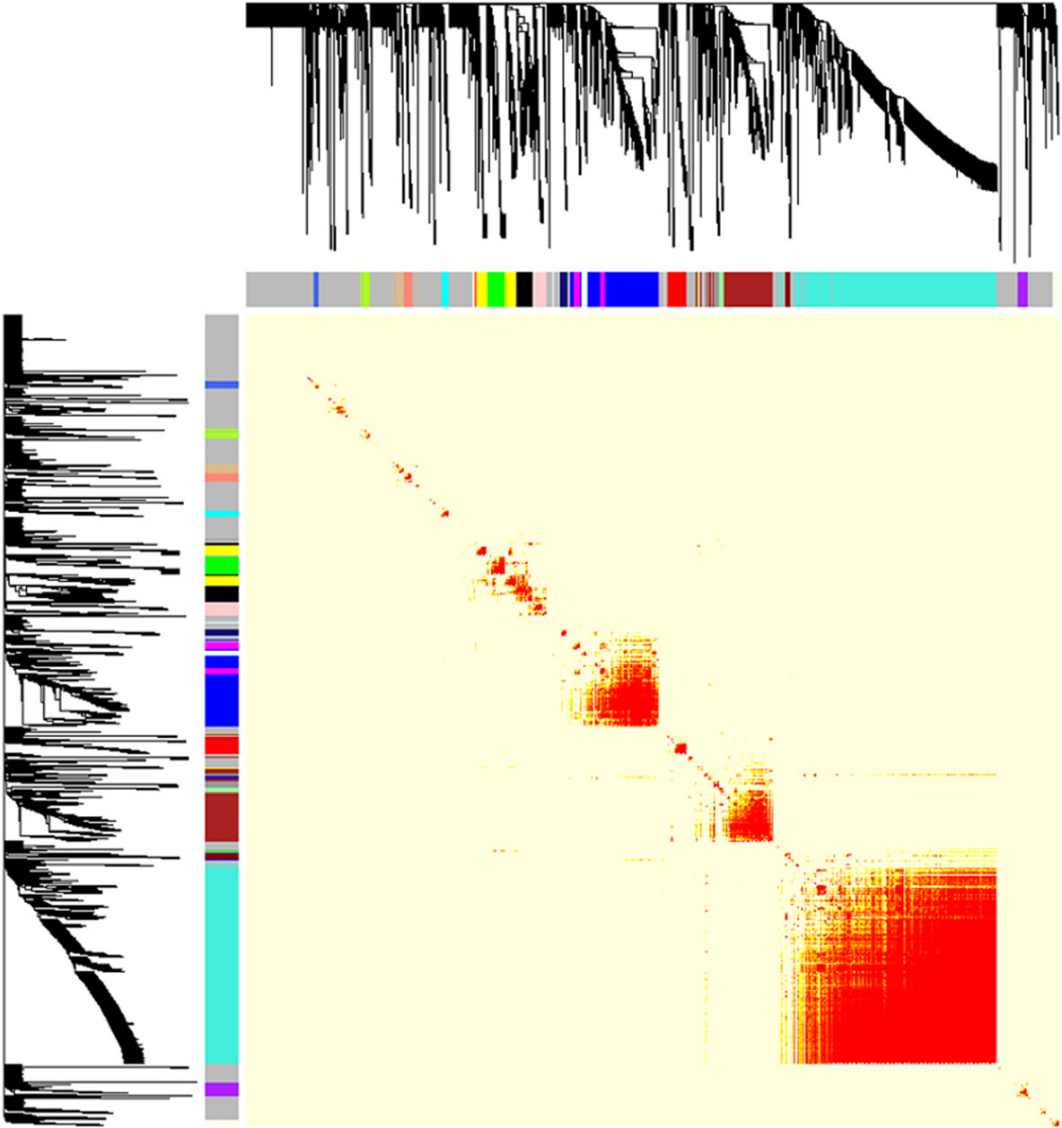
Visualizing the gene network for RNAseq dataset using a heatmap plot. The heatmap depicts the Topological Overlap Matrix (TOM) among all genes in the analysis. Light color represents low overlap and progressively darker red color represents higher overlap. Blocks of darker colors along the diagonal are the modules. The gene dendrogram with dissimilarity based on topological overlap and module assignment are also shown along the left side and the top

The WGCNA analysis identified 21 modules with a transcript number from 35 to 1244 in each module. The network heatmap plot for the transcripts was present in Fig. 3. The lncRNA-mRNA pairs with the Topological Overlap Matrix (TOM) similarity values > 0.5 were presented in Additional file 4: Table S2. An example network for ‘grey60’ module was shown in Additional file 5: Figure S3.

Overrepresentation test indicated that these mRNAs were enriched in a few biological processes, such as meiosis (*p* = 0.00164), DNA replication (*p* = 0.00246), metabolic process (*p* = 0.000838) and in molecular function, e.g., helicase activity (*p* = 0.000102) and catalytic activity (*p* = 0.0000612). The detail information of the overrepresentation test on GO-slim classification was put in Additional file 6: Table S3. The dataset may provide a basis for exploration of the lncRNA function in tilapia.

### Identification of stress-related lncRNAs

To characterize the stress-related lncRNAs in tilapia, the collected samples without replicates and stress challenges were firstly filtered. We finally kept 12 RNAseq samples in response to 24 h’s cold challenge that generated in this study and collected two published datasets under hypoxia and salinity challenges. The clean reads were first mapped to the de novo transcriptome assembly. The gene expression profiles were used in DE analysis. Twenty DE transcripts were randomly selected for validation by qRT PCR. PCR primers were successfully developed for 15 of these genes (Additional file 7: Table S4). Except the two transcripts (TCONS_00094910 and TCONS_00010892), the remaining 13 transcripts that revealed by qRT PCR showed same regulation directions as the RNAseq data (*r* = 0.73). Therefore, the qRT PCR experiments indicated a well concordance with the RNAseq expression data.

In brain under 24 h’s cold treatment, 42 DE lncRNA genes (FDR < 0.05) including 31 down-regulated and 11 up-regulated genes were identified. In heart under 24 h’s cold treatment, 28 DE lncRNA genes were observed including 6 down-regulated and 22 up-regulated genes. In gill under hypoxia challenge, 19 DE lncRNA genes (FDR < 0.05) including 13 down-regulated and 6 up-regulated were identified. In heart under hypoxia challenge, 14 DE lncRNA genes (FDR < 0.05) including 6 down-regulated and 8 up-regulated were identified. Only 2 DE lncRNA genes were found in intestines under seawater challenges. The summary statistics was put in Table 2 and the detail information was presented in the Additional file 8: Table S5.

**Table 2:** Statistics of differentially expressed lncRNAs in different tissues and/or under different stress challenges in this study

DEG types	Tissue (and stressors)	Up_DEG	Down_DEG	Total_DEG
Tissue-specific	Testis	20	0	20
	Blood	164	111	275
	Brain	37	376	413
	Gill	31	1	32
	Gonad	134	0	134
	Eye	34	0	34
	Heart	22	43	66
	Intestine	31	0	31
	Kidney	74	1	75
	Liver	118	0	118
	Ovary	219	339	558
	Skin	62	0	62
	Spleen	137	0	137
Stress-related	Brain under cold treatment	11	31	42
	Heart under cold treatment	22	6	28
	Gill under hypoxia	6	13	19
	Heart under hypoxia	8	6	14
	Intestine under seawater	1	1	2

*DEG* differentially expressed genes, *Up_DEG* up-regulated DEG, *Down_DEG* down-regulated DEG

Of the 99 unique DE lncRNA genes, only 6 simultaneously showed significant responses to two stress conditions. The lncRNA lnc_TCONS_00002141 were significantly differentially expressed in heart under cold and hypoxia treatment. lnc_TCONS_00214756 and lnc_TCONS_00290917 were significantly unregulated, but lnc_TCONS_00047189, lnc_TCONS_00226316 and lnc_TCONS_00151992 were significantly down-regulated in brain and heart under cold treatment.

We found that 5 DE lncRNA genes (lnc_TCONS_00082453, lnc_TCONS_00105572, llnc_TCONS_00144691, llnc_TCONS_00186275 and lnc_TCONS_00290917) were co-expressed with 331 mRNA genes as revealed by WGCNA network analysis. 33 of these genes (e.g., Acads, Adsl, Bard1, Calcoco2, Ccna2, Ccnb1, Cdc7, Cdk7, Cul3, Cxcr4, Dab2, Dapk1, Eif2b3, Eme1, Exo1, Fancg, Fntb, G6pd, Hnmt,Ldha, Lig4, Morf4l1, Msh2, Nfkbil1, P2rx7, Pif1, Pold3, Ppp3ca, Tbl2, Tmem8c, Tsc22d2, Ube4a, Vldlr) were mapped to the GO term GO:0006950 (response to stress).

### Tissue-specific DE lncRNA genes

We identified 1955 DE lncRNA genes with 1083 up-regulated and 871 down-regulated lncRNAs for all tested tissues. Except the DE lncRNA genes that identified in brain, heart and ovary, in which most of the lncRNAs were down-regulated, the DE lncRNA genes were absolutely up-regulated in remaining tissues, such as in spleen, skin and liver (Table 2 and Additional file 9: Table S6). We also observed many lncRNAs were shared by at least two tissues (Additional file 10: Figure S4). The distribution features was similar to previous studies that lncRNAs are frequently expressed in a tissue-specific fashion [53] and 90% of the lncRNAs are shared by at least two tissues in fish [8]. These findings indicated that the lncRNAs may regulate biological processes through their spatio-temporal expression.

### Interaction between lncRNAs and miRNAs

It is becoming increasingly evident that regulation of gene expression through lncRNAs as competing endogenous RNAs, such as competition with miRNA binding, is a general phenomenon. To characterize the interaction of miRNAs and lncRNAs, we carried out a microRNA-lncRNA interaction analysis with miRanda (ver.3) [54]. Higher alignment procedure scores based on sequence complementarity and lower minimum free energy (MFE) values reveal better thermodynamic stability of RNA duplexes [55]. We found 72,267 lncRNAs contain a motif with sequence complementary to 458 miRNAs under a strict filter parameters (miRanda total score > 120, minimum free energy <− 12 kcal/mol and matched length > =18 bp). The average score is 148.1 with a maximum score of 205. The average minimum free energy is − 17.92 (kcal/mol) with the lowest free energy of − 49.3 (kcal/mol). The 8878 highly reliable miRNA-lncRNA interaction pairs with a low free energy from − 30 to − 49.3 (kcal/mol) were provided in Additional file 11: Table S7.

The 99 stress-related DE lncRNA genes contained a motif with sequence complementary to 448 miRNAs. Ten of them contained a motif with sequence complementary to 17 mature miRNAs and a free energy less than − 30 kcal/mol. The pair of lncRNA ‘TCONS_00151992’ and miRNA ‘dre-miR-128-5p’ have the lowest minimum free energy (− 36.69 kcal/mol) and a maximum score of 175. These data suggest these miRNA molecules may involve into stress responses.

## Discussions

### Large data size of lncRNAs found in tilapia

The average sequence length of lncRNAs identified in this study was much shorter than that of the NCBI reference RNA data (an average length of 3216 bp and a N50 of 4383 bp; GCF_001858045.1_ASM185804v2_rna.fa) for tilapia, suggesting lncRNAs had shorter transcripts than protein-coding genes. This feature was generally discovered in lncRNA identification, such as in rice brown planthopper (*Nilaparvata lugens*) [56] and silkworm [57]. Besides, the exon number for lncRNAs(1.1) was much less than other types of genes(8.9)in tilapia. The finding was consistent to the previous reports, such as in Luo et al. [49] that the predicted lncRNAs contained fewer exons than protein genes. Moreover, the differences between the number of identified lncRNAs in this study and Ensemble noncoding RNAs can be due to the differences in the pipelines applied for their identification.

The human genome might harbor nearly as many lncRNAs as protein-coding genes (perhaps 15,000 lncRNA), although only a fraction were expressed in a specific cell type [58]. In the pig genome, 6621 lncRNAs were identified by analyzing 93 samples and expressed sequence datasets [59]. In *Anopheles gambiae* [60] and *Drosophila melanogaster* [61–63] around 3000 lncRNA genes were identified. In fish, 21,065 high-confident non-coding transcripts were discovered in *Salmo salar* [8] and 16,600 to 33,600 unique lncRNAs in zebrafish were found [16]. The lncRNA data size identified in the tilapia genome was more than the reported data, partially because of the large samples from different conditions and tissues used in the study.

### A few co-expression profiles for lncRNAs and mRNAs worth further investigation

The prediction of lncRNAs and mRNA interactions is very important to study the function of lncRNAs. The genes on the same pathways or in the same functional complex often exhibit similar expression patterns under diverse temporal and physiological conditions [64]. The genes show a similar expression pattern across samples and tissues can be inferred by analyzing their co-expression networks [65, 66]. The gene co-expression networks have become a rapidly developing area of study and many interesting results have been obtained, such as genes related to mouse weight [67].

In tilapia, the averaged positive correlation coefficient (*r_mean* = 0.286) between overlapped lncRNA and mRNA pairs showed significant differences with the values for all lncRNA-mRNA pairs (*r_mean* = 0.176, *z* statistics = − 2.45, *p* value = 0.00071) and mRNA-mRNA pairs (*r_mean* = 0.186, *z* statistics = − 2.23, *p* value = 0.0129). This suggests lncRNAs may have roles in cis-regulating their neighboring protein coding genes [51]. Moreover, the presence of shared regulatory elements controlling the expression of both promoters can be another possible interpretation.

We also found 55,121 pairs of lncRNA and mRNA transcripts with a TOM value range of 0.5 to 0.93. These transcripts contained 462 lncRNAs and 1023 mRNAs. A few known co-expression profiles have been found in the dataset. For example, lnc_TCONS_00284667 shows similar co-expression profiles with gene dmrt1 (TOM = 0.52) and tex11 (TOM = 0.52). Dmrt1 has been known to be involved in the process of sex determination [68]. Tex11 is associated with meiosis [69]. lnc_TCONS_00271192 is closely associated with gene slc13a1 (TOM = 0.57), slc22a13 (TOM = 0.69), slc22a7 (TOM = 0.62) and slc2a11a (TOM = 0.67). Based on earlier findings, slc13a1, slc22a6 and slc2a11 were co-expressed in human pluripotent stem cell [70]. In addition, co-expression of slc13a1 and slc22a6 was identified in transcriptional profiling of human central nervous system [71]. Hence, our study reveals closely associated relationship among genes and/or lncRNAs. However, there is little information available on the co-expression of many modulated genes. For example, lnc_TCONS_00109777 showed strong co-expression with gene enosf1 (TOM = 0.89), march7 (TOM = 0.93), cep290 (TOM = 0.93) and hps3 (TOM = 0.93). These results may worth further investigation.

### lncRNAs might play key roles in response to different stressors in tilapia

Differentially expressed (DE) analysis of the RNAseq datasets have been conducted to identify lncRNAs significantly in response to stress. For example, non-coding transcriptional response induced by pathogens has been characterized in infected salmons [8, 72] and in zebrafish under b-diketone antibiotic exposure [73].

Improvement of the stress tolerance, e.g., low-temperature, hypoxia and salt tolerance has become an important issue for aquaculture development of tilapia. The transcriptional responses to cold stress and hypoxia were explored in some teleosts [19–32]. In tilapia, the gene expression changes in response to alkalinity stress [36], salinity adaptation [37] have been carried out. These investigations have revealed a large number of stress responsible genes involved in a variety of biological processes. LncRNAs play important roles in stress responses. For example, LncRNAs TUG1 and H19 transcript levels were elevated at 1.94-fold and 2.44-fold, respectively, in skeletal muscle of hibernating ground squirrels compared with euthermicones [74]. In mice, lncRNA TUG1 exerted a protect effect against cold-induced liver damage by inhibiting apoptosis [18]. However, little information on the function of lncRNAs in cold, hypoxia and salinity stresses are available in fish. In this study, the identification of lots of differentially expressed lncRNA genes in heart, brain and intestines in response to cold stress, salt or hypoxia stress suggested that lncRNAs might play key roles in response to different stressors in tilapia.

### lncRNAs might act in miRNA function

The crosstalk between lncRNAs and miRNAs is intricate and complex. Several studies have indicated that the lncRNAs can enhance pri-miRNA processing or act as precursor [75–77]. In this study, we found 72,267 lncRNAs contain a motif with sequence complementary to 458 miRNAs. Of which, 99 stress-related DE lncRNA genes contained a motif with sequence complementary to 448 miRNAs. In addition, only low proportions of miRNA-lncRNAs pairs (~ 2%) show well complementary (100% identity) (Additional file 11: Table S7). This means very few lncRNAs may act as precursors in tilapia. Coding and non-coding RNA transcripts with shared miRNA response elements (MREs) were able to actively communicate with each other [78, 79]. Previously report indicated that when a given mRNA was up-regulated, the repression conferred by its associated targeting miRNAs was decreased, as the total number of MREs exceeds that of the miRNAs themselves [80]. However, the relationship among mRNA, lncRNA and miRNAs is still unclear. Our work provides a valuable non-coding resource to evaluate transcriptional modulation of lncRNAs under stress challenge in future.

## Conclusions

A computational pipeline based on 103 RNAseq datasets identified 72,276 lncRNAs from the tilapia genome. Co-expression network analysis identified 21 modules and important clustered genes in different tissues. DE analysis identified 99 DE lncRNA genes in response to cold, salt and hypoxia stressors and 1955 DE lncRNA genes for all tested tissues. This study provided an invaluable resource for further studies on molecular bases of lncRNAs in tilapia genomes. Further function analysis of the lncRNAs is required to elucidate their roles in regulating stress-related adaptation.

## Methods

### Fish management, cold treatment, sample collection

To identify cold stress-associated lncRNAs in tilapia, we randomly selected 36 Nile tilapia individuals (~ 90 dph) from a population raised at the fish facility of School of Life Science, Sun Yat-Sen University. These fishes were randomly divided into two indoor conical fibre glass tanks (water depth: 70 cm, volume: 500 L) in a recirculating freshwater system for acclimation of 2 days with water temperature at 25–28 °C and dissolved oxygen (DO) > 6 mg L^− 1^. The fishes were fed with tilapia pellets twice a day and the photoperiod was adjusted to 12D:12 L in the room.

During low temperature treatment, the fishes in one tank were used as the control and those in the other were considered as test samples. The temperature in the water of test tank was slowly decreased to 15 °C within 12 h using a water cooler. The control tank was maintained under 25–28 °C. Following 24 h cold treatment, the fishes were anesthetized with MS 222 before we collected six tissue samples including brain and heart from each fish. The tissue samples were kept immediately in TRIzol reagent (Invitrogen, UK) and then stored at − 80 °C.

### RNA isolation and NGS sequencing

Total RNA from cold-treated samples was isolated using TRIzol reagent (Invitrogen, UK) according to the manufacturer’s protocol. The RNA quality was assessed using the Nanodrop-2000 (Thermo Scientific, USA) and electrophoresis in 1.5% agarose Gel. Total RNA integrity was further evaluated by using Bioanalyzer 2100 (Agilent Technologies). Twelve libraries from heart and brain samples that constructed by TruSeq™RNA Sample Preparation Kit according to the product instruction (Illumina) were finally sequenced using Illumina HiSeq2500 for 2 × 150 bp pair-end (PE) sequencing.

### LncRNA identification from RNA-seq dataset

To maximize the identification of lncRNAs in tilapia genome, 12 cold–treated samples and 20 hypoxia-treated RNAseq datasets from brain, spleen, heart and gills that previously generated in our laboratory [31, 32] and other 71 RNAseq datasets downloaded from NCBI SRA database from different tilapia tissues were also collected in this study (Additional file 1: Table S1).

The RNA-seq data from each sample was first mapped to the tilapia reference genome (Orenile1.0 and Orenil1.0.88.gtf) by using TopHat [81]. The mapped reads then was de novo assembled using the program Cufflinks and Cuffmerge with default parameters [82]. The fasta sequences were generated using the program Gffread (https://github.com/gpertea/gffread) and the sequences with a length less than 300 bp were filtered. To validate the assembly, sequence similarity search of all the transcripts against 10 tilapia Transcriptome Shotgun Assembly (TSA) databases (Additional file 1: Table S1) with the basic local alignment search tool Blastn [83]. All hits with an E value <1e-6 and identity (ID) > = 98% were considered as significant blast hits.

PLEK [84] uses an improved computational pipeline based on k-mer and support vector machine (SVM) to distinguish long non-coding RNAs (lncRNAs) from messenger RNAs (mRNAs). CNCI [85] by profiling adjoining nucleotide triplets can effectively distinguish protein-coding and non-coding sequences independent of known annotations to retrieve novel lincRNAs. The lncRNAs from our transcriptome assembly were initially predicted using the software PLEK [84] with score < − 0.5 and CNCI [85] with default parameters.

Coding potential analysis of the non-coding sequences were conducted using CPC [86] and TransDecoder-3.0.0 (https://github.com/TransDecoder/TransDecoder/wiki). The candidate lncRNA datasets from the output of CPC were translated into proteins by using the software TransDecoder-3.0.0. LncRNAs contain an ORF longer than 100 amino acids were removed. The longest ORFs for the remaining candidates were then searched against Swissprot and Pfam protein database (release 27; used both Pfam A and Pfam B) (http://pfam.xfam.org/) using the program hmmscan (www.ebi.ac.uk/Tools/hmmer/search/hmmscan) and Blastp [83]. All hits with E values <1e-6 were considered as significant blast hits. The final lncRNA dataset should not have similarity to any known proteins. LncRNA candidates near gaps in the genome might represent truncated coding genes. We further filtered the lncRNA candidates that located within 100 bp of the downstream and upstream of gaps. The remaining transcripts were denoted as lncRNAs in this study (Additional file 12). The lncRNAs candidates were compared with Orenil1.0.ncrna dataset that downloaded from Ensembl genome database (www.ensembl.org).

### Genome location, distances between pairs of mRNAs and lncRNAs, and correlation of gene expression profiles

The remaining uncertain transcripts were searched against the tilapia protein database (Oreochromis_niloticus.Orenil1.0.pep.all.fa) by Blastx and mRNA_sequences from dataset GCF_001858045.1_ASM185804v2_rna_by Blastn with parameters ID > = 99% and E-value = 0. The transcripts with significant hits as revealed by both programs were considered to be mRNA candidates. The positions for lncRNAs and mRNA candidates with stranded information were then retrieved from the merged.gtf that generated using Cuffmerge. The distances for each pair of lncRNA and mRNA genes were calculated using shell scripts. The gene counts that normalized with the reads per kilobase per million mapped reads (RPKM) normalization approach were obtained by using the program Cuffnorm (http://cole-trapnell-lab.github.io/cufflinks/cuffnorm/). The correlation coefficients of gene expressions for pairwise lncRNAs and mRNAs were calculated using the R program Hmisc (https://github.com/harrelfe/Hmisc). The significance of the differences between two correlation coefficients was assessed using the Fisher r-to-z transformation using an online calculator (http://vassarstats.net/rdiff.html). The one tail *p* value and *z* statistics was provided.

### WGCNA correlation network analysis

To decrease the dataset size for network analysis, the lncRNA-mRNA pairs with |correlation coefficients| > = 0.7 were chosen for network analysis. Weighted correlation network analysis (WGCNA) including network construction, module detection, gene selection and calculations of topological properties was carried out to find clusters (modules) of highly correlated genes in the RNAseq datasets via R package WGCNA_1.49 [87]. One-step network construction and module detection methods were adopted in the analysis. A relatively large minimum module size of 30, and a medium sensitivity (deepSplit = 2) were applied for cluster splitting. The networks in each module with a TOM value > 0.5 were exported to an edge list file and plotted using the program VisANT [88]. We performed Overrepresentation test and GO annotation for mRNA gene sets using the online tool Pantherdb database (www.pantherdb.org), zebrafish genesets as reference and GO Slimmer (http://amigo1.geneontology.org/cgi-bin/amigo/slimmer). Fisher’s exact test was carried out with FDR multiple test correction and false discovery rate < 0.05. The expression heatmap was plotted using the software Genesis [89] with complete linkage mapping.

### Differential expression analysis of stress-related and tissue-specific DE lncRNA genes

To characterize the stress-related lncRNAs in tilapia, the collected samples without replicates and stress challenges were firstly filtered. Finally, we kept 12 RNAseq samples in response to 24 h’s cold challenge that generated in this study and collected two published datasets under hypoxia and salinity challenges. To identify tissue-specific DE lncRNA genes, we randomly selected 12 tissues with three RNAseq datasets for each tissue. These datasets were then used to identify tissue-specific DE lncRNA genes by pair-wise comparisons of the different tissues.

The differential expression analysis for the DE lncRNAs was performed using the tool run_DE_analysis.pl that implemented in the trinityrnaseq-2.0.6 program [90, 91] with parameters setting as --method edgeR. The significant differentially expressed lncRNAs between test samples and control samples were defined when FDR (False Discovery Rate) was less than 0.05.

### qRT-PCR analysis

qRT-PCR was performed as described in Xia et al. and Li et al. [31, 32]. Briefly, the amplifications were performed on the Roche Light Cycler 480 Real-time PCR System in a total volume of 10 μl (including 5 μl of 2 X SYBR Green MasterMix reagent, 1 μl of 1:10 diluted cDNA and 0.2 μl of each primer (10 μM)). The thermal cycling profile consisted of an initial denaturation at 95 °C for 5 min followed by 40 cycles of denaturation at 95 °C for 15 s, annealing at 60 °C for 15 s and extension at 72 °C for 20 s. An additional temperature-ramping step from 95 °C to 65 °C was used to produce the melting curve. All reactions were conducted in triplicate and using the gene EF-1α as internal control. The expression levels of genes were normalized by the levels of EF-1α in the same sample. Two-side t-test was used to compare expression levels.

### LncRNA-microRNA interaction

The RNA interaction analysis was conducted with the microRNA target prediction tool miRanda (ver.3) [54] and the dre and fru miRNA datasets that downloaded from the miRBase (www.mirbase.org). Only matched miRNA-lncRNA pairs with minimum free energy (MFE) < − 12 kcal/mol and the miRanda total scores exceeding 120 were considered as significant pairs, as suggested in Hsu et al... [55].

## Additional files

Additional file 1: **Table S1.** Summary information for RNAseq data and Transcriptome Shotgun Assembly (TSA) databases used in this study. (XLSX 18 kb)

Additional file 2: **Figure S1.** The length distribution of the lncRNAs identified in this study. (TIF 915 kb)

Additional file 3: **Figure S2.** The gene expression clustering of the RNAseq samples from 12 tissues used in the WGCNA analysis. (TIFF 1416 kb)

Additional file 4: **Table S2.** Summary for the top hub genes with TOM values more than 0.5 in modules from 12 tissue RNAseq dataset. (XLSX 2060 kb)

Additional file 5: **Figure S3.** An example showing VisANT visualization of the WGCNA weighted network. The network presents the relationships among the top hub genes in the ‘grey60” module for the RNAseq dataset. Only lncRNA-mRNA pair with TOM values > = 0.8 were presented in the network. Green circle: mRNAs; and blue circle: lncRNAs. (TIF 463 kb)

Additional file 6: **Table S3.** Overrepresentation test of the lncRNA-associated mRNA geneset in Pantherdb database. Annotation was conducted using PANTHER version 13.1 Released 2018-02-03, Fisher’s exact with FDR multiple test correction and false discovery rate < 0.05, using *Danio rerio* (REF) as background geneset. (ODS 22 kb)

Additional file 7: **Table S4.** Primer information used in the validation of DE transcript expression by qRT-PCR. (XLSX 11 kb)

Additional file 8: **Table S5.** Summary for the DE lncRNA genes detected in tilapia in response to cold, salt and hypoxia stress. (XLSX 19 kb)

Additional file 9: **Table S6.** Summary for tissues-specific DE lncRNA genes in tilapia. (XLSX 174 kb)

Additional file 10: **Figure S4.** An heatmap example showing expression changes (Log2 transformed) of partial lncRNAs among tissues as indicated by the RNAseq data. The tissue type for each sample was shown above the heatmap. (TIF 691 kb)

Additional file 11: **Table S7.** The interaction between miRNAs and lncRNA data identified in tilapia. (XLSX 575 kb)

Additional file 12: 72,276 lncRNA sequences identified in tilapia genome.rar. (FNA 56998 kb)
