# Supplementary figures and images for: Genome-wide identification and differentially expression analysis of lncRNAs in tilapia
Source: BMC Genomics. 2018 Oct 4;19:729. doi: 10.1186/s12864-018-5115-x (PMC6172845; doi:10.1186/s12864-018-5115-x)

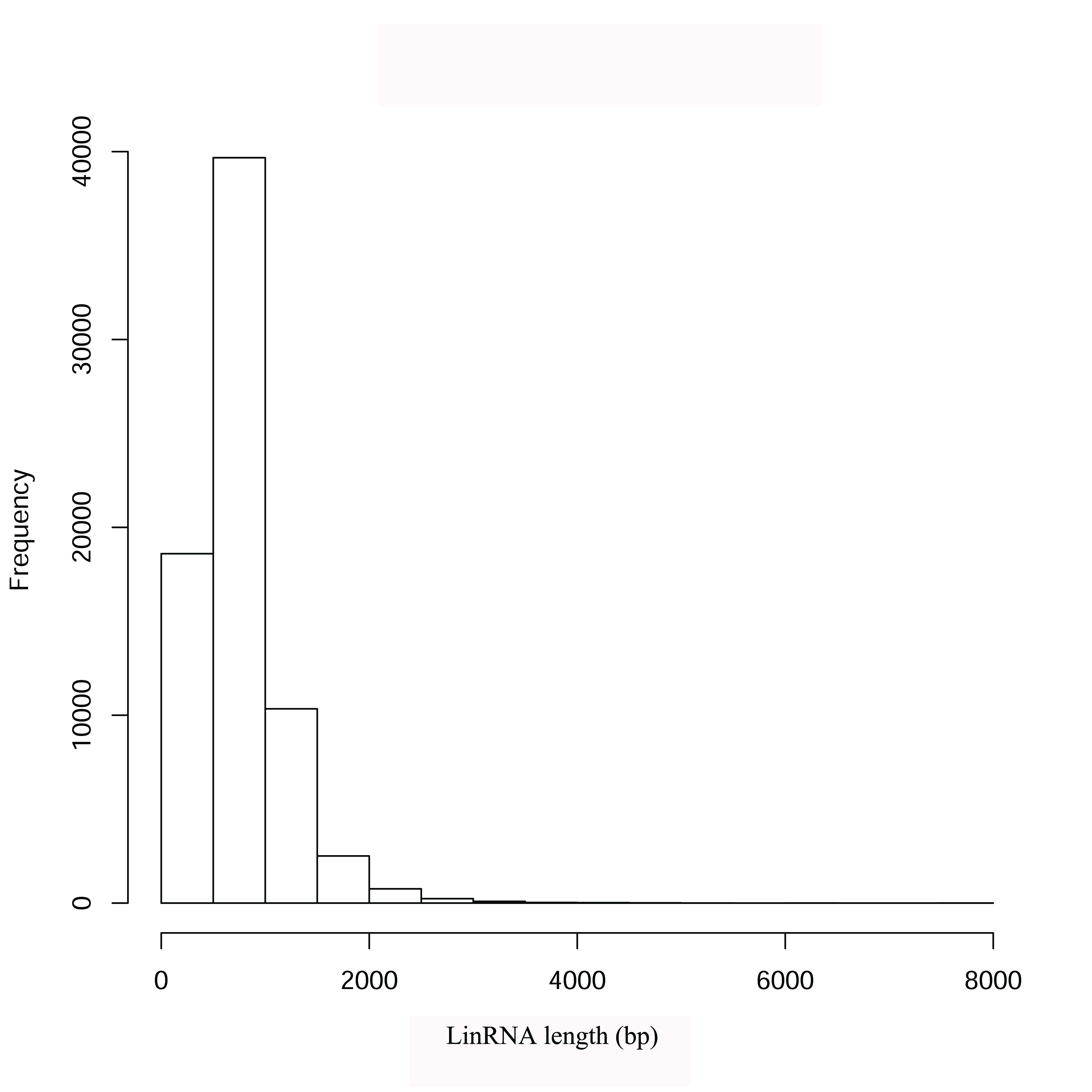

Supplement: Supplementary file 2 — Figure S1. The length distribution of the lncRNAs identified in this study. (TIF 915 kb) [file 12864_2018_5115_MOESM2_ESM.tif]

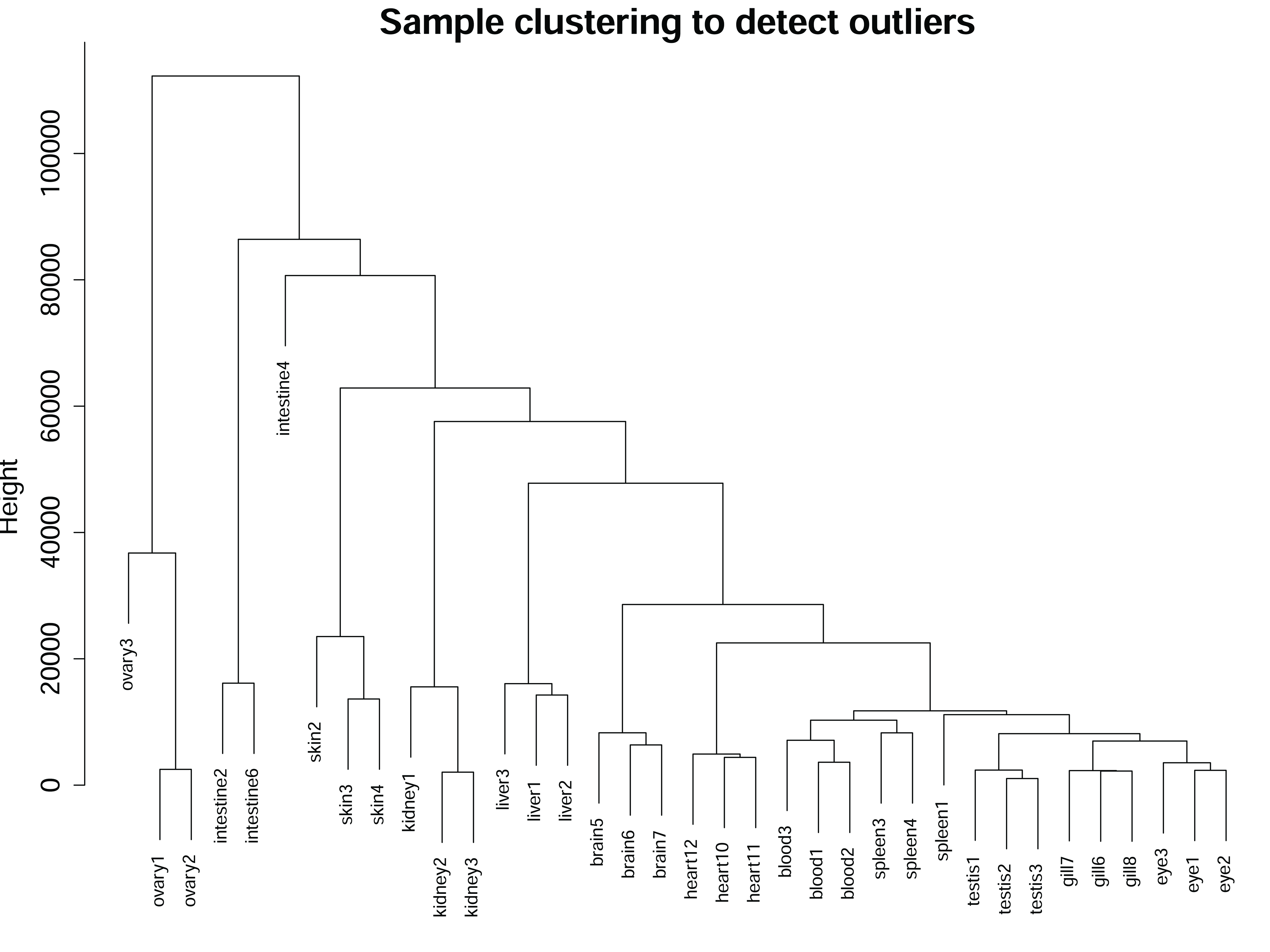

Supplement: Supplementary file 3 — Figure S2. The gene expression clustering of the RNAseq samples from 12 tissues used in the WGCNA analysis. (TIFF 1416 kb) [file 12864_2018_5115_MOESM3_ESM.tiff]

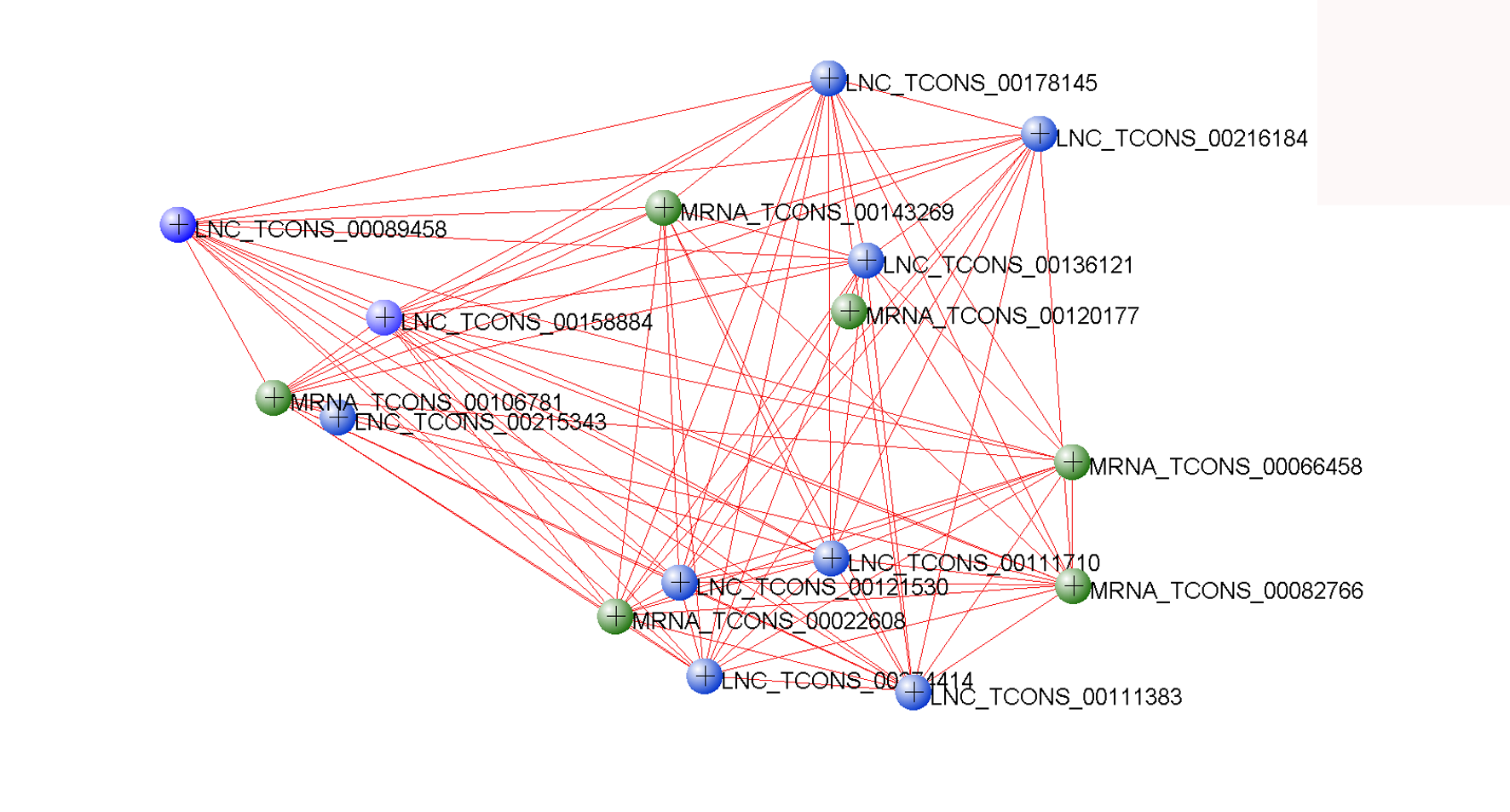

Supplement: Supplementary file 5 — Figure S3. An example showing VisANT visualization of the WGCNA weighted network. The network presents the relationships among the top hub genes in the ‘grey60” module for the RNAseq dataset. Only lncRNA-mRNA pair with TOM values > = 0.8 were presented in the network. Green circle: mRNAs; and blue circle: lncRNAs. (TIF 463 kb) [file 12864_2018_5115_MOESM5_ESM.tif]

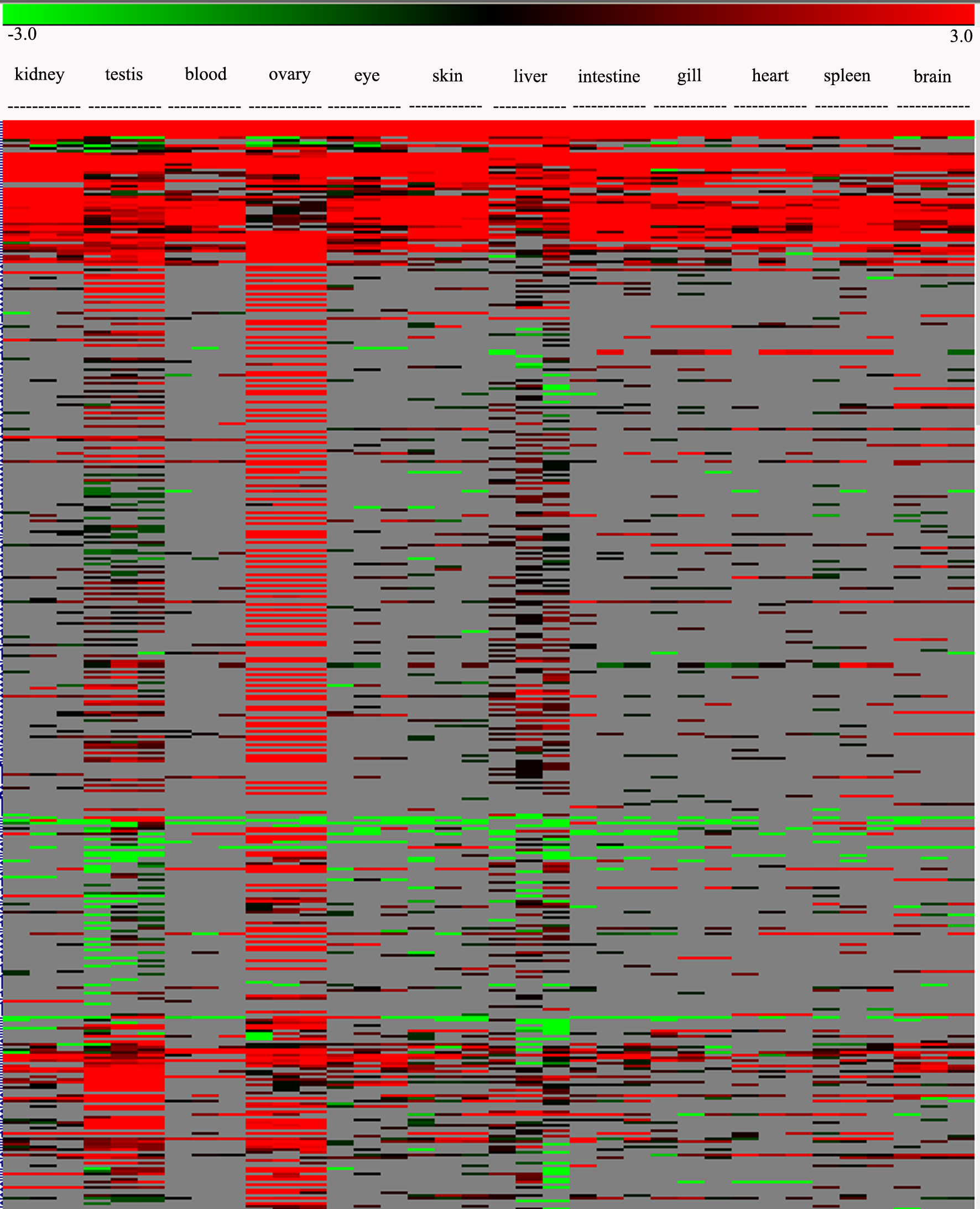

Supplement: Supplementary file 10 — Figure S4. An heatmap example showing expression changes (Log2 transformed) of partial lncRNAs among tissues as indicated by the RNAseq data. The tissue type for each sample was shown above the heatmap. (TIF 691 kb) [file 12864_2018_5115_MOESM10_ESM.tif]
